# Using Electroencephalography (EEG) Power Responses to Investigate the Effects of Ambient Oxygen Content, Safety Shoe Type, and Lifting Frequency on the Worker's Activities

**DOI:** 10.1155/2020/7956037

**Published:** 2020-04-04

**Authors:** Mohamed Z. Ramadan, Atef M. Ghaleb, Adham E. Ragab

**Affiliations:** Department of Industrial Engineering, College of Engineering, King Saud University, Riyadh, Saudi Arabia

## Abstract

**Objective:**

The study assesses the changes in electroencephalography (EEG) power spectral density of individuals in hypoxia when wearing a different type of safety shoes under different lifting frequencies. It also assesses the EEG response behavior induced via the process of lifting loads related to these variables.

**Methods:**

The study was conducted in two consecutive phases: training and acclimatization phase and experimental lifting phase. Ten male college students participated in this study. A four-way repeated measures design was used in this research with independent variables: ambient oxygen content (“15%, 18%, and 20%”), safety shoes type (“light-duty, medium-duty, and heavy-duty”), lifting frequency (“1 and 4 lifts/min”), and replication (“first and second”). And the dependent variables were alpha, theta, beta, gamma, *θ*/*α*, *θ*/*β*, *α*/*β*, *β*/*α*, (*θ* + *α*)/*β*, and (*θ* + *α*)/(*α* + *β*). The participant was allowed to determine his maximum acceptable weight of lift (MAWL) in fifteen minutes of lifting using psychophysically technique. Then, he continued lifting the MAWL for another five minutes, where all the data were collected.

**Results:**

Results showed that the EEG responses at lower levels of the independent variables were significantly high than at higher levels; except for oxygen content, the EEG responses at lower levels were considerably lower than at a higher level. It also showed that an upsurge in the physical demand increased lifting frequency and replication and caused decreasing in alpha power, theta/beta, alpha/beta, (theta + alpha)/beta, (theta + alpha)/(alpha + beta) and increasing in the theta power and the gamma power. Furthermore, several interactions among independent variables had significant effects on the EEG responses.

**Conclusion:**

The EEG implementation for the investigation of neural responses to physical demands allows for the possibility of newer nontraditional and faster methods of human performance monitoring. These methods provide effective and reliable results as compared to other traditional methods. This study will safeguard the physical capabilities and possible health risks of industrial workers. And the applications of these tasks can occur in almost all working environments (factories, warehouses, airports, building sites, farms, hospitals, offices, etc.) that are at high altitudes. It can include lifting boxes at a packaging line, handling construction materials, handling patients in hospitals, and cleaning.

## 1. Introduction

Neuroergonomics refers to the study of brain functions and structure, behavioral, and cognition output when involved in different physical and cognitive activities [1]. It is widely recognized in human factors and ergonomics (HFE) field and strives towards the development of the human-compatibility system [2]. It deals with the activity of different neurons (about 86 billion neurons) that are constantly connected through different forms of electrical signals [3, 4]. These electrical signals are a combination of brain rhythms at different frequency levels falling into differing frequency, including delta waves (<4 Hz), theta waves (4–7 Hz), alpha waves (7–12 Hz), beta waves (12–30 Hz), and gamma waves (30–50 Hz) [5]. Studies have shown that delta activities are high during sleep while theta index activities are high on the early phase of sleepiness [6]. Alpha activities reflect a relaxed attentiveness case and reductions with visual fixation, stimulation, or concentration [7]. Though, other scholars have found an increase in the activity of alpha in the drivers of the train who were sleepy sufficient to fall asleep when driving [6, 8]. Moreover, beta activity decreased during drowsiness and increased due to the attentiveness levels [9]. Torsvall [10] supposed that alpha activity was the greatest sentient measure that can be used in fatigue detecting, followed by theta and delta activities. Nevertheless, the activity of delta was more related to the appearance of sleep proper. These activities are also reported to impact the neuromuscular activities of an individual [11].

Controlling motor activities and muscle movements is the function of the brain [12, 13]. Studies in physical neuroergonomics confirm that the cerebral cortex is contributing to smoothing high-speed motor control processes and muscle activation [12, 14]. Electroencephalography (EEG) is the most common method that used the technique of neuroimaging in neuroergonomics [1]. Electroencephalography is a noninvasive technique where the electrodes were placed on the scalp to measure electrical activities for the human brain [5]. Parasuraman and Rizzo [15] proposed that monitoring of the brain for ergonomic research requires high-temporal resolution and must be economical, sensitive, robust, and unobtrusive. The electroencephalography (EEG) method meets all of these principles and helps in an efficient analysis of the capacities in a more challenging real-life condition [16].

Several methods that have been suggested to detect fatigue using electroencephalography (EEG), for instance, detection of alpha spindles [17], a process that uses the combination of all frequencies components of electroencephalography (EEG) to signify alertness level [18]. Most researchers [19, 20] suggest that classifiers can be developed for categorization of the signals that consider the individual characteristics and can be robustly applied in a controlled laboratory setting.

Studies [12, 21] have highlighted two algorithms ([*θ* + *α*/*β* and *β*/*α*) for the detection of fatigue. The delta activity is not included in the study due to its reflection of the human sleeping case. Earlier research of Eoh et al. [9] supposed that the (*θ* + *α*)/*β* is a credible fatigue indicator as it detects the increase in the ratio between the fast wave and slow-wave activities. Similarly, Jap et al. [22] reported two algorithms (*θ* + *α*)/(*α* + *β*) and *θ*/*β* for the detection of fatigue. However, the detection of fatigue has not been applied to manual working, such as lifting, pulling, and carrying, which today accounts for the five primary causes of injury [23]. Previous work that has been carried out in the discipline has been central to the medical caretakers and drivers, indicating a research gap [24, 25].

“The amount of oxygen in the air indicates molecules of oxygen present in the air per volume unit that decreases as the altitude increases. Furthermore, the pressure of atmospheric significantly affects the functions of the human body because low atmospheric pressure leads to a decrease in partial oxygen pressure [26]. Thus, the change in atmospheric pressure and the molecular pressure of oxygen are essential variables affecting oxygen transport and human respiration in altitude [27]. Many studies have shown that the maximal workload, as well as the uptake of oxygen, decreases as the atmospheric pressure and the partial pressure of oxygen decreases (in another word as altitude increases) [28, 29]”.

And if the oxygen reaching to the cells is scanty, “the hydrogen will interact with the pyruvic acid and convert it into lactic acid. This temporary anaerobic metabolism produces a small amount of energy. The accumulation of lactic acid in the blood and tissue indicates that there is insufficient amount of oxygen in the mitochondria, which can be due to hypoxia or lack of blood flow (such as shock) or a mixture between them [30], If this is prolonged or severe, it can lead to cell death. Moreover, during activity, performance is determined by the amount of oxygen transferred by the circulatory system to the muscles [31]. And muscle fatigue occurs when the intensity of the activity is high due to a lack of oxygen enough to get the metabolism and the accumulation of inorganic phosphate [32, 33]”. Sudden exposure to high altitude leads to increased ventilation as well as heart rate, “mainly driven by hypoxemia-induced carotid chemoreceptor activation, sympathoexcitation and vagal withdrawal” [34, 35], “as the increase in heart rate compensates for the lack of oxygen. And when a person stays in high places for several days, resting heart output normalizes because of the lowering in the volume of stroke due to the decrease of plasma volume because of the hypoxia [36].” However, “after acclimatization, the heart rate and Ventilation, continue increased by a sustained sympathetic response driven by peripheral chemoreceptors sensitized by the persistent hypoxemia” [37, 38].

Additionally, “few studies have evaluated the effect of shoes during lifting tasks. Aghazadeh and Lu [39] studied the impact of changing the body's position by wearing flat shoes, 5 cm, or 7.6 cm while performing floor to knuckle and knuckle to shoulder lifts at maximum lifting capacity. These findings suggest that high-heeled shoes might impact lifting and back injuries. Li et al. [40] studied the influence of footwear and floor slipperiness conditions on material handling workers who work in various types of footwear. They concluded that there is a significant effect of friction level on a perceived sense of slipping, the maximum acceptable holding weight, oxygen uptake (VO_2_), and efficiency of energy. Kim et al. [41] examined the kinematics and characteristics of the lower extremity and trunk using electromyography in the sit-to-stand (STS) job when wearing different high-heeled shoes (1, 4, or 8 cm). The results showed a significant difference in muscle activity among the different high-heeled STS conditions”.

Al-Ashaik et al. [23] “assessed the lifting capabilities of individuals while wearing various safety shoe types in a warm environment and examined the physiological responses during lifting tasks. They concluded that the safety shoe type influenced the physiological responses of the human body. The study findings demonstrated the need to account for the type of safety shoes worn, which is a safety requirement by most employers when calculating the recommended weight limits. There is a scarcity of research on the integrated impact of the discussed variables.” Ghaleb et al. [42] assessed the lifting capabilities of individuals when wearing different types of safety shoes and concluded that the safety shoe type did not significantly affect HR. Krings et al. [43] studied the effect of wearing tactical boots and steel-toed work boots, as required safety guidelines, on oxygen consumption and cardiorespiratory responses during walking. They recommended that manufacturers improve footwear design by reducing footwear mass to decrease energy expenditures.

From the previous studies, there were many measurements used to evaluate worker behavior during lifting tasks, for example, EMG, ECG, and rating of perceived exertion. But still, there is limited research that exists on the physiological demands of lifting tasks at different altitudes and lifting frequencies while wearing safety shoes. Also, there is a lack of knowledge of how EEG responses associated with lifting tasks since no study in the literature exists as the authors know. Thus, the objective of this study is to assess the changes in electroencephalography (EEG) power spectral density of individuals while wearing different safety shoes in three different levels of hypoxia under different lifting frequencies. It also examines the EEG responses produced by the lifting tasks associated with the mentioned factors. Moreover, this psychophysical study examines the following hypothesis: “the oxygen content, worn safety shoes, lifting frequency, and replication have a significant effect on EEG responses”.

## 2. Methods and Materials

### 2.1. Study Design

The experimental study design was used for assessing the changes in electroencephalography (EEG) power spectral density of individuals in different ambient oxygen content when wearing a different type of safety shoes under different lifting frequencies. Then, a quantitative approach was applied for the statistical representation of the results, which are easy to understand and comprehend. The study design was similar to the one used in an established previous research [23].

### 2.2. Participants

The study population consists of the male students enrolled at King Saud University. The random sampling method was used for recruiting ten participants, (aged 24–33 years (mean [*SD*]: 26.3 [2.53] years), mean height [*SD*] 165.1 [3.01] cm, mean shoulder height [*SD*] 138.7 [2.90] cm, mean knuckle height [*SD*] 67.55 [2.03] cm, and mean weight [*SD*] 66.63 [8.04] kg) as per the inclusion criteria. Before experimenting, the participants were prohibited from smoking and consuming tea and caffeine for approximately four hours. Siepmann and Kirch (2002) pointed out that caffeine and tea have to be excluded from EEG studies because they have “significant reduction of total EEG power at fronto-parieto-occipital and central electrode positions of both hemispheres when the subjects kept their eyes open” [44]. The medical checkup was held for ensuring that participants did not have any problem concerning skeletal, respiratory, or circulatory systems. The researchers obtained approval from the university's institutional review board by submitting a written copy of the study sample and procedure (E-19-4247).

### 2.3. Apparatus

Experiments were performed to study the electroencephalography (EEG) characteristics of workers working in the lifting tasks on different altitudes. “All experiments were done inside the environmental chamber made in (Weiss Technik UK LTD, Loughborough, UK). The environmental chamber is self-controlled of air content concerning percent of oxygen content, dry-bulb temperature, and relative humidity inside the chamber, and presented all concerned data in a display. In addition, SENSIT® P400 Multi-Gas Monitor was used to gauge the ambient oxygen content before collecting data at any session.” “An eight-channel Biomonitor ME6000, MT-ECG-1 preamplifier, EEG Amplifier 4ch for ME6000, and Mega Win 3.0.1 software (Mega Electronics Ltd., Kuopio, Finland)” were used to record the EEG signals. Besides, EMOTIV EEG headset was used for holding the EEG electrodes in place, and headphones were used to reduce the noise inside the chamber. Each used instrument was initially calibrated and checked based on the manufacturers' recommendations before each testing session. Two-handle box (60 cm × 40 cm × 22 cm) was used for lifting the loads. Also, three types of safety shoes manufactured by Shelterall Company (Italy) were used.

### 2.4. Experimental Design

**Figure 1:**
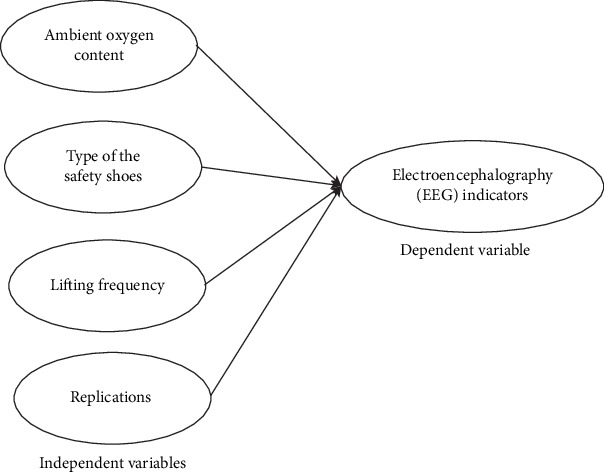
Study variables.

The independent variables in this study were ambient oxygen content, type of safety shoes, and lifting frequency. Ambient oxygen content frequency levels were “15%, 18%, and 21%”; the types of safety shoes were “light, medium, and heavy” while lifting frequency levels were 1 and 4 lifts/minute. The EEG indicators were dependent variables, as shown in Figure 1. Two replication analyses were conducted. EEG signals were recorded from the right side of the head at F4, in which the frontal cortex is in charge of executive functions, and C4, in which the central cortex is in charge of for the control of voluntary motor movement. Ground and reference electrodes were placed on the forehead and over the mastoid region (behind the right ear) of the participant, respectively. The placed electrodes were following the international 10–20 systems (Andreassi, 1995). “A significance level was set to 0.05, and factors such as ambient oxygen content or safety shoe type identified as having a significant effect on the dependent variables were further analyzed using Post-hoc tests to identify what levels of the factor are different in their effect on the dependent variables. In addition, if an interaction was found to have a significant effect on the dependent variables, a simple effect technique was conducted to demonstrate the effect at each level of one of the independent factor” [45].

### 2.5. Experiments Procedures

Training and acclimatization sessions were performed for 14 days (2 hours per day) before the experiment. “It was done to familiarize the participants with the conditions of the experiment. Training is necessary to develop participant's muscles and lifting capabilities and to become more representative of workers in the industrial environment and also that training reduces the impact of learning effect later when conducting the experimental lifting phase. When the participants were ready and acclimatized to the new environment, the procedure for data collection began. Each participant was asked to lift the weight from knuckle to shoulder. Each participant did the lifting under each of the 18 experimental conditions in 18 days (one experimental condition a day), because there are three independent variables, i.e., oxygen content environments (“21%, 18%, and 15%”) (These are equivalent to attitude 0, 1500 and 2760 meters above sea level, and these altitudes represent low, moderate and high altitude, respectively [46].” These procedures were carried out for nearly 98% of the world's population lives at 2,500 meters or less [47], lifting frequency (“1 and 4 lift/min”) [48], and safety shoes (“light-duty, medium-duty and heavy-duty; and each one was in different size so that each participant wore the size that suits his foot.” The definition of the shoe type comes from manufacturing. For more details about the safety shoe type, please see study [23]), where Table 1 shows the specifications for different types of safety shoes and Table 2 shows the 18 experimental conditions and those done randomly.

Prior to conducting the lifting task, each participant was equipped with placed EEG electrodes on the head. Then, five-minute EEG signal artifacts were recorded (artifacts include resting with a closed eye, eye blinking, chewing, body motion [arm, leg, and head movements], and physical task [lifting]). These signals were used during data analysis to remove artifacts from the EEG signals; after which, each participant performed the lifting task within the chamber under every 18 conditions of the experiment. The lifting was performed from knuckle to shoulder in the sagittal plane with no twisting using the freestyle method, and the horizontal distance was closed to the participant's body as possible. Participants lifted a two-handle box (40 cm × 60 cm × 22 cm) having the weights inside the enclosed box. After lifting the box to the shoulder level, assistance was provided for bringing the box back to the knuckle level.

**Figure 2:**
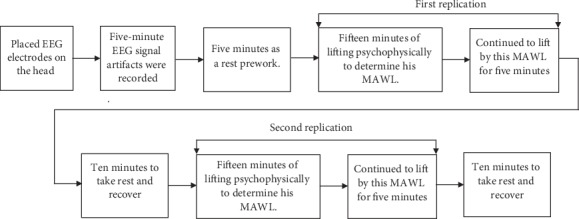
Experiments procedures after acclimatization.

**Figure 3:**
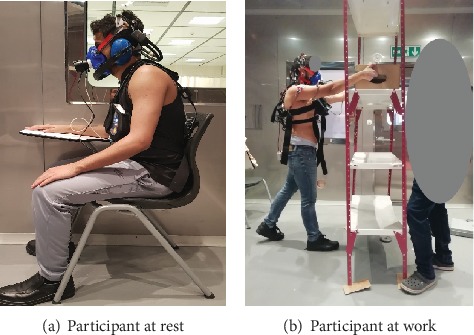
Participant at rest and at work.

Every participant was allowed five minutes as a rest prework and then followed by fifteen minutes of lifting psychophysically to determine his MAWL [49]. (“In the psychophysical approach, a person adjusts the load such that repetitive lifting does not result in overexertion or excessive fatigue. The weight selected by the subject is referred to as the MAWL”). After affirmation of the participant MAWL for the experimental session, the participants continued to lift by this MAWL for five minutes. Next, in these five minutes of experimental lifting, the participant is allowed ten minutes to take rest and recover (within the chamber) followed by a second replication of lifting for another twenty minutes similar to the first replication (fifteen minutes of lifting psychophysically to determine his MAWL. After affirmation of the participant MAWL for the experimental session, the participants continued to lift by this MAWL for five minutes). Afterward, in the second replication, the participant was provided five minutes rest to recover inside the chamber.  Figure 2 shows the experiment's procedures after acclimatization. The difference between the two replications was in their starting weights. Such as for one replication, it started with 30% of 1RM (where 1RM is the maximum amount of weight that a person can possibly lift for one repetition), while in the other, it started from no weight, which was randomly done for every session. During the five minutes of the two replications for lifting using the maximum acceptable weight of the lift, the EEG signals were measured and recorded.  Figure 3 shows the participant at rest and at work.

## 3. Data Analysis

### 3.1. Data Acquisition

The electroencephalography EEG measuring device was an “eight-channel Biomonitor ME6000, MT-ECG-1 preamplifier, EEG Amplifier 4ch for ME6000, and Mega Win 3.0.1 software (Mega Electronics Ltd., Kuopio, Finland) at a sampling rate of 1000 Hz.” “Matlab 2015b and Statistical Package for Social Sciences (SPSS) Version 22” were used to preprocess and analyze the data.

### 3.2. Preprocessing

The EEG raw data were contaminated with noise, which was removed through a preprocessing procedure. Independent components analysis (ICA) was used to remove nonstationary high-variance signals from the recorded raw EEG signals and to rebuild the missing data with a spatial mixing matrix. This algorithm uses a 5-minute recording of EEG during resting with a closed eye, chewing, body motion (arm, leg, and head movements), eye blinking, and physical task (lifting). A preprocessing on the EEG signal was carried out to remove undesired signals (noise). Subsequently, a low-pass four-pole elliptic filter with a cutoff frequency of 50 Hz was used to remove the power line noise and any high-frequency noises.

### 3.3. Data Reorganization

The preprocessed data were divided into *δ* band 0-4 Hz, *θ* band 4-8 Hz, *α* band 8-13 Hz, *β* band 13-30 Hz, and *γ* band 30-50 Hz. The *δ* band 0-4 Hz was not involved in this study, because it occurs in a deep sleep case and commonly overlaps with artifacts.

### 3.4. Indices

The EEG indices were classified into two clusters: the relative index and the ratio index, which were obtained from the reorganized data. The relative index, i.e., relative *α*, is calculated as

(1) $$\begin{array}{c} \text{Relative power }\alpha =\frac{\alpha }{\alpha +\theta +\beta +\gamma }. \end{array}$$

Since the basic indices have a tendency to “contradict each other,” the ratio indices were calculated to amplify the difference [9]. The ratio indices that used in this study were *β*/*α*, *θ*/*α*, (*θ* + *α*)/*β* [22, 50, 51], (*θ* + *α*)/(*α* + *β*), *θ*/*β*, and *α*/*β* [22].

### 3.5. Analysis

The data were processed using “Matlab 2015b, while the collected data were analyzed using IBM SPSS version 22.0 (IBM). A four-way repeated measures design with four independent variables was used. Therefore, the experiment had 36 conditions upon combinations of levels of the independent variables. The least significant difference method was used for pairwise comparisons of the main effects to identify significantly different levels of the main variables. In addition, if an interaction had a significant effect on the dependent variables, a simple effect technique was conducted to demonstrate the effect at each safety shoe type and ambient oxygen content factors [45]. The Shapiro–Wilk test was implemented to test data normality [52]. The statistical significance was set at a confidence level of 95%”.

## 4. Results

### 4.1. The Basic Indices

#### 4.1.1. Relative Alpha Power *α*

The results showed that the lifting frequency had a significant effect only on the alpha power at the central cortex, *F*(1, 9) = 5.172, *p* < 0.049. The alpha power at lifting frequency of 1 lift/min was significantly higher (mean, *SD* = 0.251, 0.1) than at lifting frequency of 4lift/min (mean, *SD* = 0.223, 0.058). However, the independent variables did not have a significant effect on alpha power at the frontal cortex.

#### 4.1.2. Relative Theta Power *θ*

**Figure 4:**
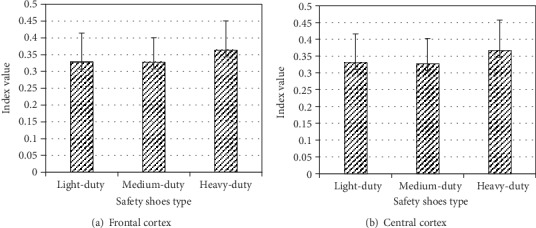
Effect of safety shoe type on relative theta power at the frontal and central cortex.

The main variable safety shoes type and three-way interaction between ambient oxygen content, lifting frequency, and replication had significant effect on the theta power at the frontal cortex (*F*(2, 8) = 16.605, *p* < 0.001, *F*(2, 8) = 5.073, *p* < 0.038, respectively) and at central cortex (*F*(2, 8) = 11.57, *p* < 0.004, *F*(2, 8) = 4.601, *p* < 0.047, respectively). At frontal and central cortexes, the theta power at heavy safety shoes was significantly higher than at light and medium safety shoes (Figure 4). However, there is no significant effect between light and medium safety shoes.

**Figure 5:**
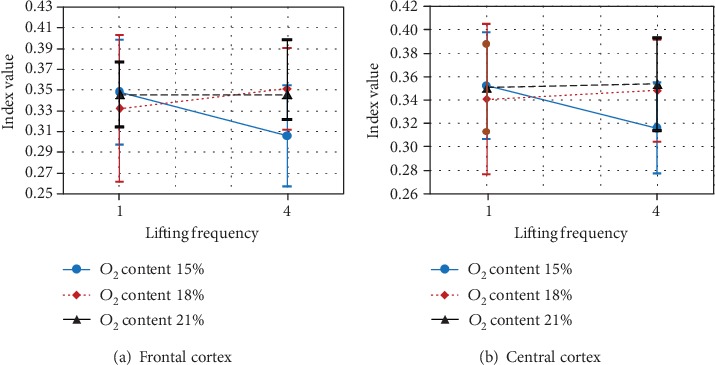
Effect of ambient oxygen content by lifting frequency by first replication on relative theta power at frontal and central cortexes.

In addition, the simple effect technique was used to analyze ambient oxygen content by lifting frequency by replication interaction (Figure 5). The results showed that at the first replication, the theta power of the frontal and central cortex was significantly lower while doing 4 lifts/min and ambient oxygen content 15% when compared with ambient oxygen content 18% or 21% (Figure 5).

**Figure 6:**
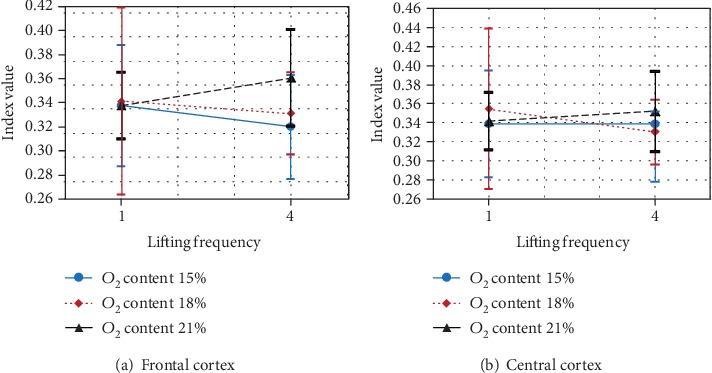
Effect of ambient oxygen content by lifting frequency by second replication on relative theta power at frontal and central cortexes.

Finally, the simple effect technique was used to analyze ambient oxygen content by lifting frequency by replication interaction (Figure 6). The results showed that at the second replication, the theta power of the frontal cortex was significantly lower while doing four lifts/min and ambient oxygen content 15% than when ambient oxygen content 21%. However, the theta power of the central cortex was significantly lower while lifting frequency 4 lifts/min and ambient oxygen content 18% than when ambient oxygen content 21% (Figure 6).

#### 4.1.3. Relative Beta Power *β*

**Figure 7:**
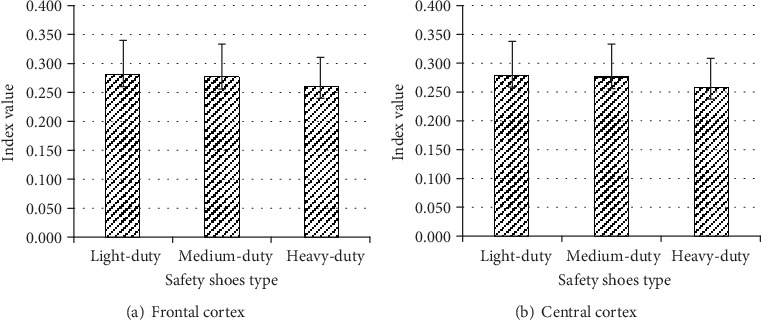
Effect of safety shoe type on relative beta power at the frontal and central cortex.

The main variable safety shoes type only had significant effect on the beta power at the frontal cortex (*F*(2, 8) = 4.817, *p* < 0.042) and at central cortex (*F*(2, 8) = 6.397, *p* < 0.022). At frontal and central cortexes, the beta power at heavy safety shoes was significantly lower than at light safety shoes (Figure 7). However, there is no significant effect between light and medium safety shoes or between heavy and medium safety shoes.

#### 4.1.4. Relative Gamma Power *γ*

**Figure 8:**
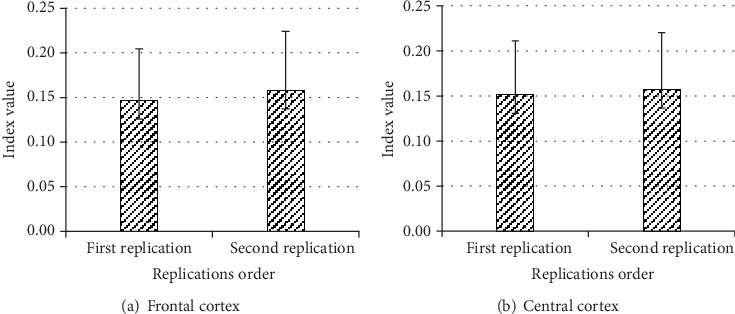
Effect of replication on relative gamma power at the frontal and central cortex.

The replication only had significant effect on the gamma power at frontal and central cortex, *F*(1, 9) = 11.492, *p* < 0.008; *F*(1, 9) = 35.332, *p* < 0.0001, respectively). At frontal and central cortexes, the gamma power at first replication was significantly lower than at second replication (Figure 8).

### 4.2. The Ratio Indices

#### 4.2.1. Theta/Alpha

The results show that in the central cortex, just the safety shoe type causes a significant effect on the theta/alpha (*F*(2, 8) = 4.824, *p* < 0.042). However, in the frontal cortex, the safety shoes type and two-way interaction between safety shoes type and replication had significant effect on the theta/alpha (*F*(2, 8) = 4.545, *p* < 0.048) and (*F*(2, 8) = 4.816, *p* < 0.042), respectively.

**Figure 9:**
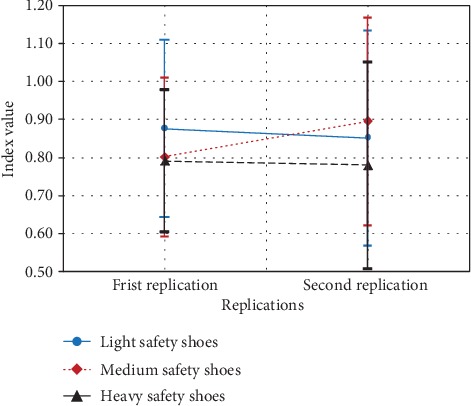
Effect of safety shoes type by replications on theta/alpha at frontal cortex.

The simple effect technique was used to analyze safety shoe type by replication interaction (Figure 9). The results showed that at the frontal cortex, the theta/alpha was significantly lower while wearing heavy safety shoes than light safety shoes at first replication, and the theta/alpha was significantly lower while wearing heavy safety shoes than light or medium safety shoes at second replication (Figure 9).

#### 4.2.2. Theta/Beta

The factor that has significant effect on theta/beta at central cortex was frequency lifting only *F*(1, 9) = 5.459, *p* < 0.044. The theta/beta was significantly less when lifting frequency was 4 lifts/min (mean [*SD*] = 0.88 [0.35]) when compared to 1 lift/min (mean [*SD*] = 1.189 [0.79]). Furthermore, three-way interaction between ambient oxygen content, safety shoes type and replication, and four-way interaction between ambient oxygen content, lifting frequency, safety shoes type, and replication had significant effect on the theta/beta at frontal cortex, *F*(4, 6) = 4.83, *p* < 0.0445, and *F*(4, 6) = 6.83, *p* < 0.02, respectively.

**Figure 10:**
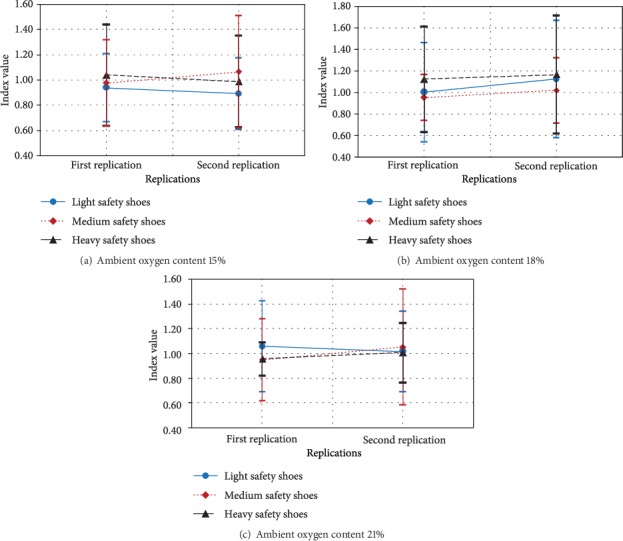
Effect of ambient oxygen content by safety shoe type by replications on theta/beta at the frontal cortex.

Generally, the simple effect technique was used to analyze ambient oxygen content by safety shoe type by replication interaction. The results show that at ambient oxygen content 15%, the theta/beta was significantly lower at the second replication while wearing the light safety shoes than while wearing the medium safety shoes, as shown in Figures 10(a) and 10(b) shows that at ambient oxygen content 18%, the theta/beta was significantly lower at first replication when wearing the medium safety shoes than when wearing the heavy safety shoes.

#### 4.2.3. Alpha/Beta

In the central cortex, the results show that the safety shoe type had a significant effect on the alpha/beta (*F*(2, 8) = 4.613, *p* < 0.047), where the alpha/beta at heavy safety shoes was significantly higher than at medium safety shoes. However, there is no significant effect between light and medium safety shoes or between light and heavy safety shoes. In additional, at frontal cortex, the safety shoes type and three-way interaction between safety shoes type, lifting frequency, and replication had significant effect on the alpha/beta *F*(4, 6) = 4.83, *p* < 0.044 5 and *F*(4, 6) = 6.83, *p* < 0.02, respectively, where the alpha/beta at heavy safety shoes was significantly higher than at light or medium safety shoes. However, there is no significant effect between light and medium safety shoes.

**Figure 11:**
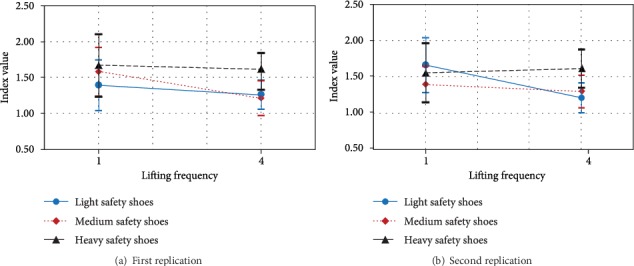
Effect of lifting frequency by safety shoe type by replications on alpha/beta at the frontal cortex.

Furthermore, as shown in Figure 11(a), at the first replication, the alpha/beta was significantly higher while doing 4 lifts/min and wearing the heavy safety shoes than when wearing the light or medium safety shoes. Finally, at the second replication, the alpha/beta was significantly higher while doing 4 lifts/min and wearing the heavy safety shoes than when wearing the light or medium safety shoes (Figure 11(b)).

#### 4.2.4. Beta/Alpha

The safety shoes type only had significant effect on the beta/alpha at frontal and central cortexes (*F*(2, 8) = 5.869, *p* < 0.027) and (*F*(2, 8) = 10.428, *p* < 0.006), respectively. However, there is no significant effect between the types of safety shoes in the frontal and central cortex although the means of beta/alpha in the frontal cortex were 1.219, 1.064, and 0.959 and in the central cortex were 1.172, 1.054, and 0.904 while wearing the light, medium, and heavy safety shoes, respectively.

#### 4.2.5. (Theta + Alpha)/Beta

The lifting frequency only had significant effect (theta + alpha)/beta at the central cortex, *F*(1, 9) = 6.726, *p* < 0.029. The (theta + alpha)/beta at lifting frequency of 4 lifts/min was significantly lower (mean, *SD* = 2.23, 0.49) than at lifting frequency of 1 lift/min (mean, *SD* = 2.79, 1.05). However, there is no any factor effecting on (theta + alpha)/beta at frontal cortex.

#### 4.2.6. (Theta + Alpha)/(Alpha + Beta)

The results show that there is no any variable had significant effect on (theta + alpha)/(alpha + beta) at frontal cortex. But vice versa at central cortex, the two main variables ambient oxygen content and lifting frequency had significant effect on (theta + alpha)/(alpha + beta), *F*(1, 9) = 6.582, *p* < 0.020 and *F*(1, 9) = 5.207, *p* < 0.048, respectively. The (theta + alpha)/(alpha + beta) for participants at lifting frequency of 4 lifts/min was significantly lower than the (theta + alpha)/(alpha + beta) at 1 lift/min. Also, the (theta + alpha)/(alpha + beta) for participants at ambient oxygen content of 15% was significantly lower than the (theta + alpha)/(alpha + beta) at either ambient oxygen content of 18% or ambient oxygen content of 21%.

## 5. Discussion and Conclusion

This research studied the EEG activity in different frequency bands, classified into two clusters: the relative indices (alpha, theta, beta, and gamma) and the ratio indices (*θ*/*α*, *θ*/*β*, *α*/*β*, *β*/*α*, [*θ* + *α*]/*β*, and [*θ* + *α*]/[*α* + *β*]), at different brain cortex to evaluate the efficiency of these indices as methods to detection of fatigue. The results of the experiments for relative indices presented significant differences between the lifting frequencies at central cortex for alpha, safety shoes types, and interaction between ambient oxygen content, frequency, and replication at frontal and central cortexes for theta, safety shoes types at frontal and central cortexes for beta, and replications at frontal and central cortexes for gamma. These results are similar to the previous studies results which conclude that decreased in the power of the numerous bands of EEG at central and frontal cortexes related to increase the workload [53–55].

The experiment showed that EEG power of theta decreased as the ambient oxygen content decreased at frontal and central cortex. This finding is consistent with other researches that reported decreased of the alpha activities is one of the most features of cerebral hypoxia [54]. Accordingly, some researchers also found an increasing alpha activity during fatigue [6, 56]; on the contrary, the alpha, beta, and gamma increased as the ambient oxygen content decreased at frontal and central cortex, but these decreases and increases in the brain signal were not significant because participants were subject to acclimatization sessions for 14 days. Furthermore, hypoxia leads to excessive ventilation and thus to hypocapnia of arteriolar. This situation may account to regional cerebral hypocapnic vasoconstriction, with changes in lethargy, the flow of cerebral blood, and synchronization of EEG [57]. Belyavin and Wright [58] reported an increase in theta and delta activities at fatigue; other studies reported an increase of theta at fatigue [9]. Lal and Craig [59] also showed a significant increase in theta and delta activity; however, the activity of beta and alpha at fatigue showed a smaller increase. Nevertheless, Eoh, Chung, and Kim [9] reported that the increase in activity of alpha is the most sensitive indicator of fatigue.

The experiment results in the present study for ratio indices reveals significant differences between the safety shoe types at central cortex for theta/alpha, as well as safety shoe type and two-interaction between safety shoes types and replications at frontal cortex for theta/alpha, also frequencies at central cortex for theta/beta and three-interaction between ambient oxygen content, safety shoes, and replication and four-interaction between ambient oxygen content, lifting frequency, safety shoes, and replication at frontal cortex for theta/beta. Moreover for theta/beta, the finding showed significant differences between safety shoes at central cortex and safety shoes and three-interaction between frequency, safety shoes, and replication at the frontal cortex. Also, there were significant differences between the type of shoes at frontal and central cortexes for beta/alpha, as well as between frequencies at central cortex for (theta + alpha)/beta. Finally, ambient oxygen content and lifting frequency had significant differences on (theta + alpha)/(alpha + beta) at the central cortex.

Hence, a combination of several indices in detecting fatigue might be required to enclose the reliability of the method that can be used for the detection of fatigue. Therefore, take into consideration that safety shoes can be a stress-adding variable, which might add to the acceleration of the fatigue state transition and where no fatiguing conditions are present. The study acknowledges the fatigue markers recorded in the physiological responses. The findings also suggest that the EEG monitoring should also follow some reforms in the regulation such criteria for the selection of the shoes, the type of shoe as per the service nature to ensure adequate protection. Accordingly, periodic breaks between the works should also be ensured to optimize the performance of individuals and lower the detrimental impact that can occur. And the applications of these tasks can occur in almost all working environments (factories, warehouses, airports, building sites, farms, hospitals, offices, etc.) that are at high altitudes. It can include lifting boxes at a packaging line, handling construction materials, handling patients in hospitals, and cleaning.

## Figures and Tables

**Table 1:** Specifications for safety shoes.

	Heavy-duty safety shoes	Medium-duty safety shoes	Light-duty safety shoes
Upper	Waxy full grain leather	Genuine full leather with double density PU sole	Full leather with double density PU sole
Linings	Cambrele woven	Cambrele	Cambrele
Tongue	Padded	Padded	—
Lacing	Through 4 pairs eyelets	Through 4 pairs eyelets	—
Collar	Padded	Padded	Padded
Toe caps	Steel (toe cap)	Steel (toe cap)	Steel
Sole	Polyurethane	Polyurethane moulded	Rubber
Innersole	Full sock	Full sock	Full sock
CUT	High-cut	Low-cut	Low-cut
Addition	Steel plate	—	—

**Table 2:** Eighteen combinations for independent variables.

No.	Conditions
1	A1B1C1
2	A1B1C2
3	A1B2C1
4	A1B2C2
5	A1B3C1
6	A1B3C2
7	A2B1C1
8	A2B1C2
9	A2B2C1
10	A2B2C2
11	A2B3C1
12	A2B3C2
13	A3B1C1
14	A3B1C2
15	A3B2C1
16	A3B2C2
17	A3B3C1
18	A3B3C2

A1 is the oxygen content environments 15%, A2 is the oxygen content environments 18%, A3 is the oxygen content environments 21%, B1 is the light safety shoes, B2 is the medium safety shoes, B3 is the heavy safety shoes, C1 is the lifting frequency 1 lift/min, and C2 is the lifting frequency 4 lifts/min.

## Data Availability

The data used to support the findings of this study are available from the corresponding author upon request.
